# Evaluation of Lateral Incisor Resorption Caused by Impacted Maxillary Canines Based on CBCT: A Systematic Review and Meta-Analysis

**DOI:** 10.3390/children9071006

**Published:** 2022-07-05

**Authors:** Anastasia Mitsea, Georgia Palikaraki, Konstantinos Karamesinis, Heleni Vastardis, Sotiria Gizani, Iosif Sifakakis

**Affiliations:** 1 Department of Oral Diagnosis & Radiology, School of Dentistry, National and Kapodistrian University of Athens, 11527 Athens, Greece; amitsea@dent.uoa.gr; 2 Department of Orthodontics, School of Dentistry, National and Kapodistrian University of Athens, 11527 Athens, Greece; gewpalhkarakh@gmail.com (G.P.); k.karamesinis@gmail.com (K.K.); vastard@dent.uoa.gr (H.V.); 3 Department of Paediatric Dentistry, School of Dentistry, National and Kapodistrian University of Athens, 11527 Athens, Greece; stgizani@dent.uoa.gr

**Keywords:** impacted canines, root resorption, systematic reviews, meta-analyses, radiography, orthodontics

## Abstract

Background: Root resorption (RR) of the adjacent teeth due to upper canine impaction requires an appropriate modification of the orthodontic treatment plan and the mechanotherapy used. Aim: The aim of this review was to assess scientific evidence published during the last decade, concerning the prevalence of lateral incisor RR caused by impacted maxillary canines, based only on cone-beam computed tomography (CBCT). The location of RR on this tooth, as well as the prevalence of RR on the other adjacent teeth, were additionally evaluated. Methods: This review followed the criteria specified by the PRISMA statement. Four databases were searched for articles published between January 2008 and June 2021. Predefined and piloted data collection forms were used to record the necessary information. Results: A total of 5098 records were initially screened. Only seven articles were finally eligible for further analysis. A total number of 540 participants (176 males and 364 females) was derived from the included studies. RR of maxillary lateral incisors was common (50%). RR of mild severity was more common (62%), more frequently located in the middle (52%) and apical (42%) thirds of the root. Conclusions: Further research with more homogeneous groups is required.

## 1. Introduction

Maxillary canines are the most frequently impacted teeth, following third molars [1]. The incidence of maxillary canine impaction ranges from 1.7% to 5.4% and differs between different populations [1,2,3,4,5,6] and genders [1,5]. Bilateral impaction occurs in 8% of these cases [1,6]. In addition, 85% of the impacted maxillary canines are located palatally. This anomaly is less common in the mandible (0.35%) [5,7,8,9].

The maxillary canine bud is located high in the maxilla, lateral to the piriform fossa, above the root of the lateral incisor, and remains there until the calcification of its crown. As a result, its eruption path is longer and more tortuous compared to other teeth [10]. One-third of the root development in a maxillary canine is reached by the age of 8.5–10.5 years. Moreover, an early sign of canine impaction is overlapping with a lateral incisor in panoramic radiographs if the development of the lateral incisor is already complete [11]. The etiological factors of impaction may be local or systemic [10,12]. The role of heredity is indicated by a concurrence of other dental anomalies, a frequent bilateral occurrence, a different incidence between genders or races/ethnicities, and a high familial occurrence [13].

Early detection of canine impaction is critical since this anomaly may lead to several complications. Internal or external root resorption (RR) of the impacted canine may occur but the most common complication is RR of the adjacent teeth. Cases with extended lesions are not rare, even with pulp involvement of the adjacent lateral and/or the central incisor [14]. Furthermore, loss of vitality, displacement, canine ankylosis, follicular cysts as well as recurrent infections and pain may be observed. Additionally, a loss of space and shortening of the dental arch perimeter may occur. Severe cases present with several of these complications [15,16,17].

Early detection of the aberrant canine development may significantly reduce the risk of these complications and especially the incidence/extent of RR. Traditionally, two-dimensional (2D) radiographs are used for early diagnosis. The limitations of these images, i.e., superimposition, distortion, projection, and the inability of detecting RR less than 0.6 mm in diameter and 0.3 mm in depth, should be always considered [18,19]. Cone-beam computed tomography (CBCT) is a more precise and accurate imaging method compared with conventional radiographs for the localization of impacted teeth and RR; however, it should be used only when the information provided by conventional radiography is inadequate [18,19,20]. Herein, the present systematic review aimed to assess scientific evidence published during recent decades concerning the prevalence of lateral incisor root resorption (RR) caused by impacted maxillary canines, based only on cone-beam computed tomography (CBCT). The teeth most frequently affected as well as the location of RR were additionally assessed.

## 2. Materials and Methods

### 2.1. Design

The present systematic review was written according to the Preferred Reporting Items for Systematic Reviews and Meta-Analyses (PRISMA) statement [21]. Computerized literature research with language restrictions was conducted in June 2021 by two of the authors independently (G.P. and K.K.).

### 2.2. Eligibility Criteria

Eligibility criteria were based on the PICOS (Participants, Intervention, Comparison, Outcomes, Study design) framework. The following inclusion criteria were implemented: studies evaluating RR of impacted maxillary canines with CBCT, articles in the English language published during the last decade, and patients with a free medical history and permanent teeth.

#### 2.2.1. Types of Participants

The evaluated studies were conducted in healthy patients with no age limits and at least one impacted maxillary canine.

#### 2.2.2. Types of Exposure

Studies evaluating RR of lateral incisor caused by maxillary impacted canines.

#### 2.2.3. Types of Outcome Measures

The primary outcome measure was the prevalence of lateral incisors’ RR. The secondary outcome measures were the location of RR and severity of lateral incisors’ RR. The influence of patients’ ethnicity and age was considered secondary. The severity of root resorption was evaluated according to the Ericson and Kurol (2000) grading method [14]:

- severe resorption (resorption reaches the pulp),
- moderate resorption (resorption of the dentine midway to the pulp or more, the pulp lining being unbroken),
- slight resorption (resorption of less than half the dentine thickness),
- no resorption (intact root surface).

### 2.3. Study Design

The present review was limited to studies that used CBCT in any field of view. Articles using CBCT with a combination of panoramic, medical computed tomography, or other radiography techniques were not included. The following publications were excluded: case reports/case series, reviews (systematic and literature), personal opinions, in vitro studies, author debates, letters to the editor, author responses, books and/or book chapters, abstracts, editors’ summaries, congress abstracts, summary articles, non-English articles, and articles evaluating panoramic or conventional radiographs.

### 2.4. Search Strategy

The searched databases included Scopus, PubMed, Science Direct, and Cochrane Library. A determined search was performed to identify any relevant studies based on various combinations of keywords. The aforementioned electronic databases were searched for articles published between January 2008 and June 2021 using the following keywords: “impacted maxillary canines”, “cone beam computed tomography”, “lateral resorption”, “CBCT”, and “resorption”. The Boolean operators “AND” and “OR” were used to enhance the search strategy through several combinations. Articles in languages other than English were excluded. In addition, efforts were made to obtain additional or ongoing trials from the reference lists of the eligible studies and relevant reviews. The authors of the studies were contacted if additional clarifications were needed.

The process for selecting studies was the following: (A) two of the authors (G.P. and A.M.) systematically and independently analyzed the titles and selected the articles whose titles met the objectives of this study. (B) The same reviewers systematically analyzed the abstracts of the selected articles by applying the inclusion and exclusion criteria. Article titles that met the objectives of the study but did not have abstracts available were fully analyzed in the final evaluation. (C) Full texts of the eligible studies were obtained and evaluated to verify whether they fulfilled the eligibility criteria. Disagreements between the authors were discussed carefully before the final decision. The excluded studies were registered separately, clarifying the reasons for rejection. The reference lists of all retrieved full-text articles were fully searched for relevant articles. (D) Finally, the articles that didn’t answer the clinical research questions were excluded.

### 2.5. Data Collection and Data Items

Data extraction was performed independently by the same two authors (G.P. and A.M.) and in duplicate. All disagreements were resolved by discussion or consultation with the help of a third author (K.K.). Predetermined and pre-piloted data collection forms were used to record the necessary information (total number of patients, ethnicity, number of males and females, age [mean and SD], total number of impacted canines, and number of bilateral and unilateral impacted canines [total left/right] in males, females and in total, severity, and location of RR).

### 2.6. Risk of Bias in Individual Studies

The risk of bias was assessed using the Risk Of Bias In Non-Randomized Studies of Interventions (ROBINS-I) tool [22]. Two authors (G.P. and A.M.) assessed the risk of bias in individual studies, both independently and in duplicate. Disagreements between the authors were discussed carefully to reach a consensus. However, if the two authors could not reach a consensus, the article was forwarded to a third author (K.K.) for the final decision on quality ratings.

### 2.7. Risk of Bias across Studies

Several factors may have affected the cumulative evidence in the present meta-analysis. All studies included patients that were referred for impacted canines without taking into account systematic errors derived from the measurement method and ethnicity. Additionally, differences existed in voxel sizes, fields of view, CBCT scanning protocols, age distributions as well as in patient selection. Expertise in reading CBCT images and the quality of the CBCT images may have also affected the detection and evaluation of RR.

### 2.8. Summary Measures and Synthesis of Results

The primary outcome was lateral incisor RR caused by impacted maxillary canines. The location and severity of RR were evaluated as well as its correlation with the angulation of the impacted canines, age, and gender.

A random-effect model [23] was used for pooling proportions using the metaprop command in STATA, which was developed for performing meta-analyses of binomial data [24]. The pooled prevalence of lateral incisor RR caused by impacted maxillary canines and the corresponding 95% confidence interval (CI) are provided, as well as the pooled prevalence of lateral incisor RR by severity and location. Heterogeneity across studies was further assessed using the Q test and the I2 metric [25]. Stata version 13.1 (Stata Corp., College Station, TX, USA) was used for statistical analysis and the significance level was set at 5%.

The overall strength of evidence was assessed after considering the following parameters of the studies and their findings:

- quality (assessment of individual studies),
- consistency (the extent of similarity between different studies in their findings) and
- quantity (number of studies, magnitude of treatment effect, sample size across studies).

## 3. Results

### 3.1. Study Selection

**Figure 1:**
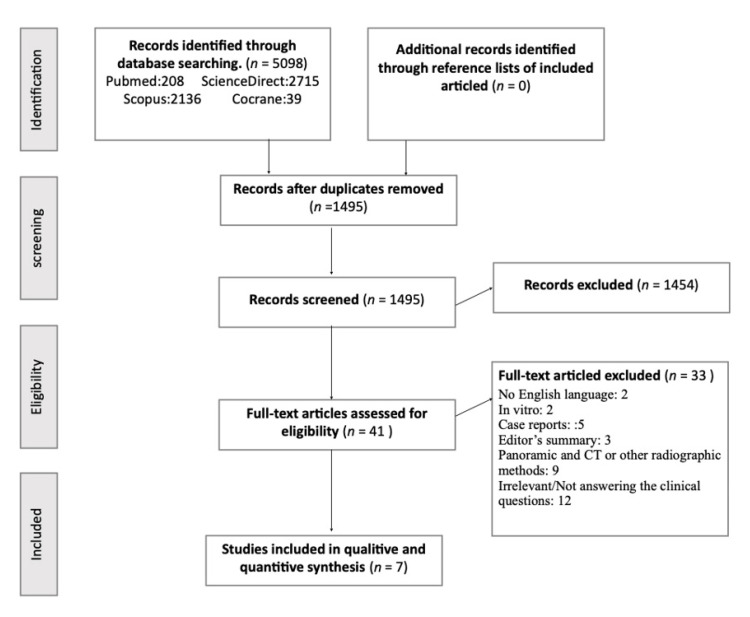
Flow diagram.

The results of the literature search, identification, inclusion, and exclusion of the articles are presented in the flow diagram according to the PRISMA statement (Figure 1). The electronic and manual search initially identified 5098 relevant records, whereas 1495 remained after a manual duplicate check. Forty-one articles were selected for a full review according to the inclusion and exclusion criteria after the title and abstract screening. Nine of them used only CT or a combination of CT and panoramic radiographs. Five of them were case reports, whereas two were in vitro studies. Two were written in a language other than English, another three were editors’ summaries, and the remaining twelve papers did not answer the clinical question. Consequently, seven articles were identified and included in the qualitative and quantitative synthesis [26,27,28,29,30,31,32].

### 3.2. Description of Studies

The general characteristics of the seven studies, as well as the sample characteristics, are depicted in Table 1. The total of 540 participants (176 males and 364 females) was derived from the included studies. The selected studies used only CBCT in order to evaluate the impacted canines and RR. All seven studies evaluated the severity of RR of lateral and central incisors [23,24,25,26,27,28,29,30], five of them evaluated RR of the first premolar [26,27,28,30], and three of them studied RR of the second premolar [22,24,27]. Additionally, the location of the lesion was studied in four papers [27,29,30,31].

### 3.3. Risk of Bias in Individual Studies

Table 2 depicts the risk of bias for the eight included studies [26,27,28,29,30,31,32]. All of them were assigned an overall risk of bias in terms of moderate risk.

### 3.4. Risk of Bias across Studies

In the majority of the included studies, the main methodological problem was the absence of a control group [26,27,28,29,30,31,32]. Only one study [29] evaluated a control group of patients with normally erupted canines.

### 3.5. Results of Individual Studies

Seven studies were identified that could be included in the meta-analysis. The number of impacted canines in each study ranged from 42 to 210 and RR affected the lateral or/and central incisors and/or premolars. RR in the lateral incisors was evaluated in all studies. All studies evaluated the severity of the resorption (severe, moderate, or slight) using Ericson and Kurol’s (2000) grading method [14]. Four out of the seven studies evaluated the location of the RR on the adjacent tooth (apical, middle, or cervical one-third) [27,28,29,30].

**Figure 2:**
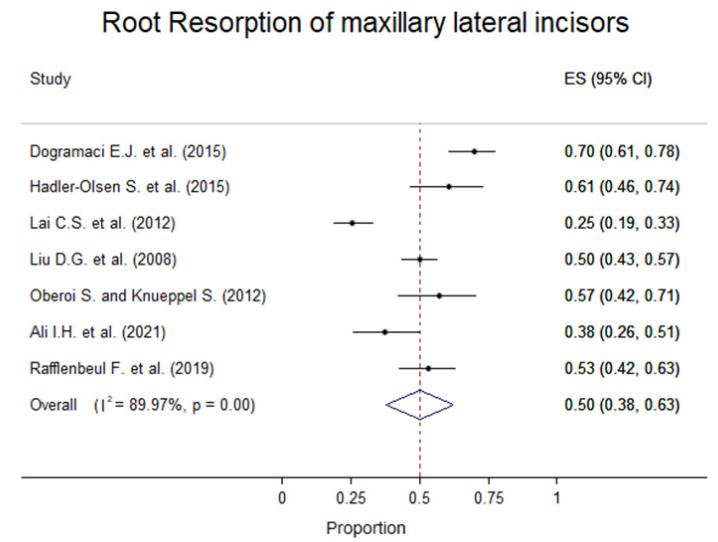
Forest plot with the prevalence of root resorption on patients with impacted canine [26,27,28,29,30,31,32].

Different prevalence values of impacted canines in central and lateral incisors were found ranging from 4.0% to 46.67% for central incisors and 30.09% to 96.00% for lateral incisors. The prevalence values for RR in the premolar incisors ranged from 0% to 23.88%. The root resorption of maxillary lateral incisors ranged from 25% to 70% in the different studies (Figure 2).

**Figure 3:**
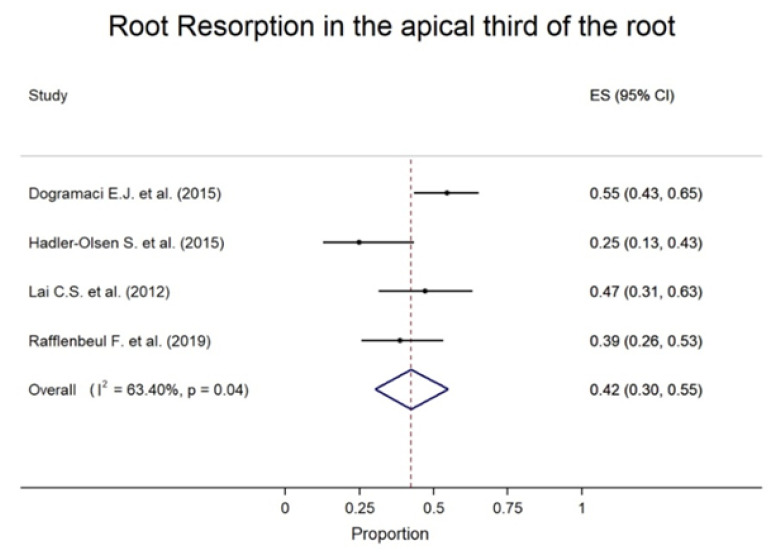

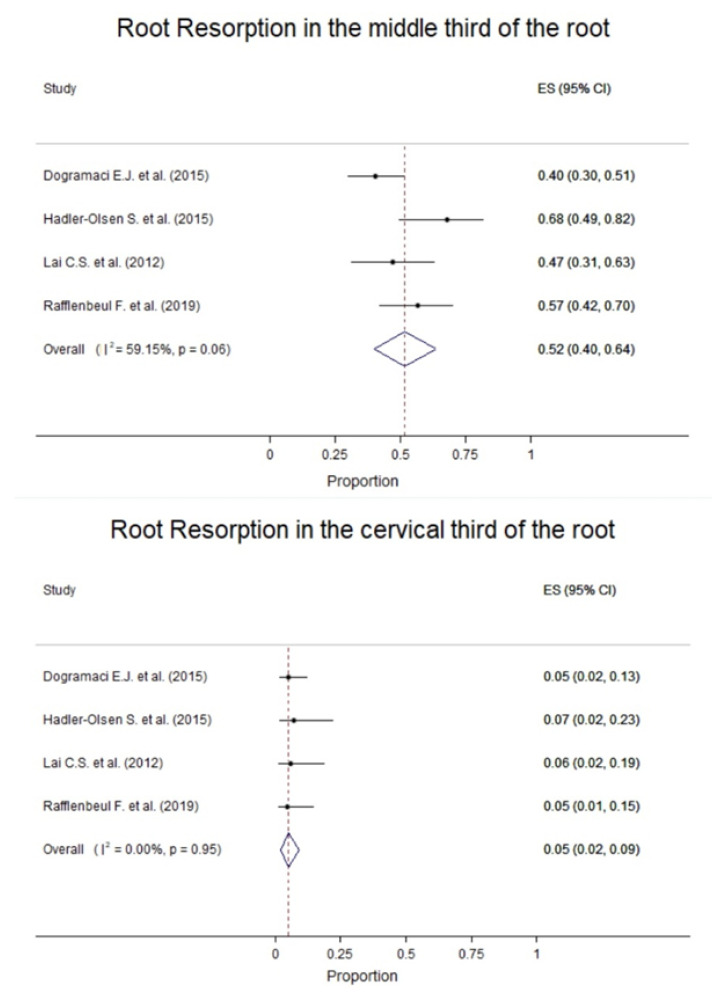
Forest plots with the prevalence of root resorption for patients with impacted canines by location [26,27,28,29,30,31,32].

**Figure 4:**
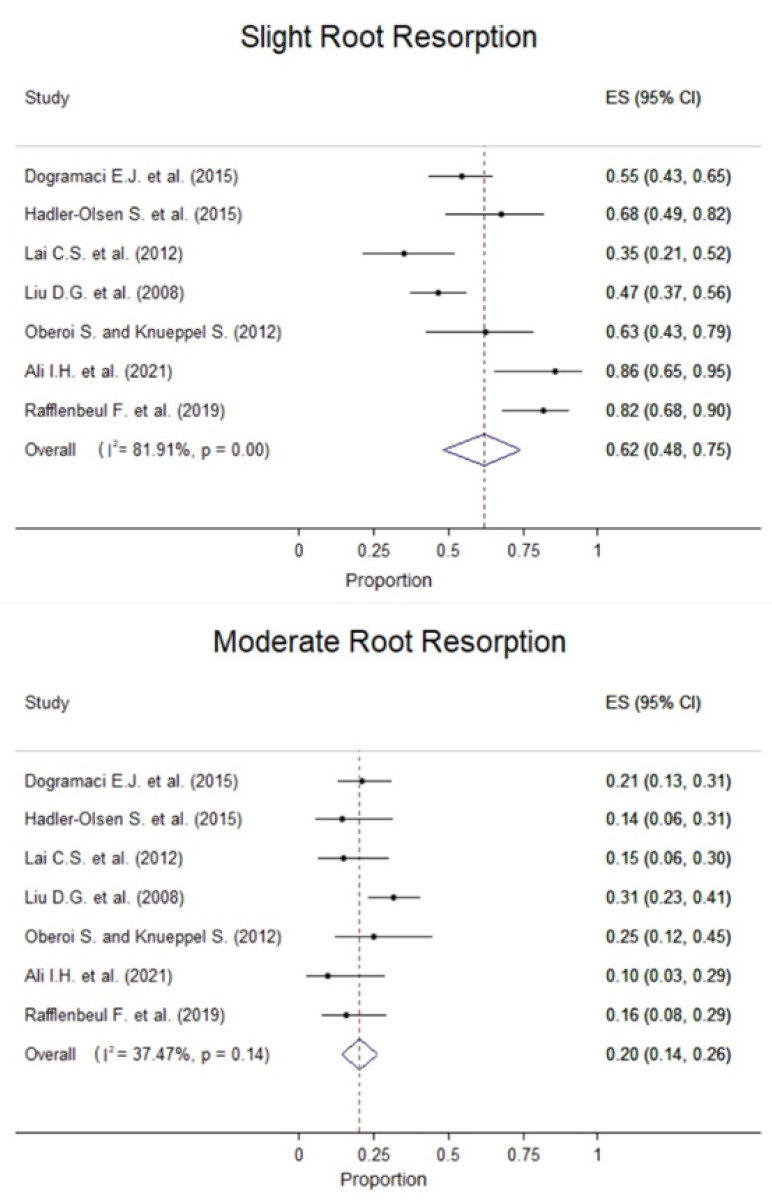

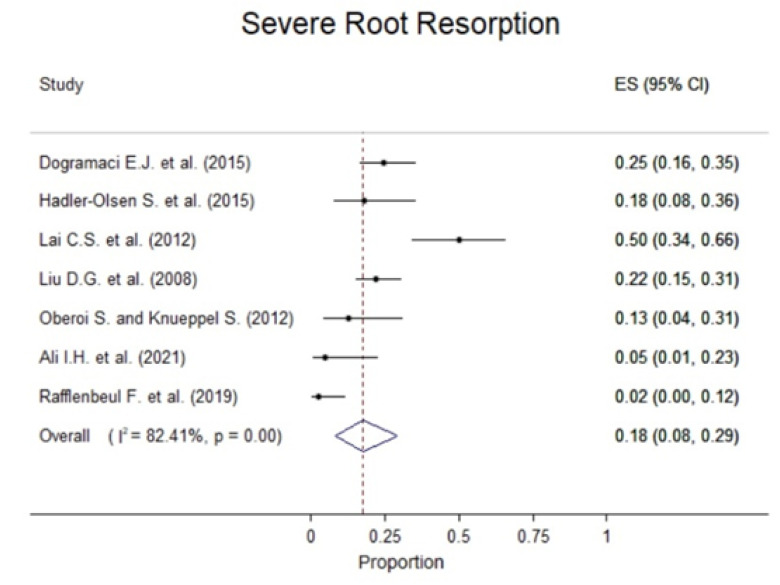
Forest plots with the prevalence of root resorption for patients with impacted canines by severity [26,27,28,29,30,31,32].

Regarding the location of the RR on the adjacent tooth, the middle one-third was the most prevalent and the cervical one-third was the least (Figure 3). Regarding RR severity, mild resorption was the most common, ranging from 35.29% to 85.71%, although severe RR was demonstrated in 2.27% to 50.00% of cases across the studies (Figure 4).

### 3.6. Synthesis of Results

The prevalence of RR was higher in the lateral incisor with an overall prevalence of 66% (95% CI: 50–80%), followed by the central incisor [18% (95% CI: 7–31%)] and the premolar (7% [95% CI: 1–15%)].

The results from the meta-analysis revealed that the pooled prevalence of root resorption in lateral incisors was 50% (95% CI: 38–63%). The I2 statistic value was 89.97% (*p*-value < 0.05), supporting high statistically significant heterogeneity between the study-specific prevalence of root resorption (Figure 3).

Pooled from four studies, RR was most commonly reported in the apical one-third with an overall prevalence of 42% (30–55%) between the resorbed teeth (Figure 4). In most cases, the severity of RR was slight with a pooled proportion of teeth with slight resorption of 62% (95% CI: 48–75%) and the pooled proportion of teeth with moderate resorption was 20% (95% CI: 14–26%). In teeth with severe resorption, the pooled proportion was 18% (95% CI: 8–29%) (Figure 4). The severity and location of root resorption showed generally high between-study heterogeneity; however, this should be carefully interpreted because of the small number of studies.

No sensitivity analysis was performed, since after excluding the studies with a moderate risk of bias, no study remained for the analysis in both meta-analyses.

## 4. Discussion

CBCT in some cases of maxillary displaced canines is highly beneficial in clinical decision making regarding the prognosis of the canine and adjacent teeth, the proper access for the surgical approach, and the direction of orthodontic traction. These cases mainly concern the presence or suspicion of RR. This is a frequent complication that remains challenging for orthodontists since it requires an appropriate modification of the treatment plan and the mechanotherapy used [31,32]. Recently, a systematic review was published evaluating this issue [33]. The authors of this review included studies that used either only CBCT or a combination of panoramic and CBCT. However, the accuracy of detecting RR differs according to the radiographic technique [20,33]. In the present study, the literature was systematically reviewed in order to evaluate the extent to which lateral incisor RR is caused by impacted maxillary canines by solely applying CBCT in order to minimize bias across studies and obtain maximal homogeneity.

CBCT provides more accurate imaging than conventional 2D radiographs. The decreased accuracy of RR diagnoses using the latter technique may be attributed to the superimposition of the incisor and the canine as well as to the magnification errors and distortions due to root angulation, commonly observed among 2D imaging techniques. These inherent disadvantages may lead to an underestimation of the extent of the lesion. RR in the early stages cannot be diagnosed using 2D imaging unless the entire root surface is resorbed buccolingually to the point that alters its projection mesiodistally [5,18,34]. Moreover, it is not possible to comparatively evaluate the root thickness between successive exposures. On the contrary, 3D techniques not only detect the presence of RR in all dimensions, but also track the position of the impacted tooth accurately. CBCT allows for a more precise evaluation of RR, especially in cases of minor dentin lesions [15,34,35]. High accuracy and sensitivity for RR detection were found in several CBCT systems [35]. The present study summarizes the results of seven retrospective studies that used CBCT scanning images to evaluate the RR of the adjacent teeth due to the impaction of maxillary canines. RR of the lateral incisors was found to be rather common (50%); however, in most cases (62%) the resorption was mild. The lesion was most frequently located in the middle (52%) and apical (42%) thirds of the root. It should be emphasized that significant variance was observed among the selected studies regarding the incidence of RR. It was not possible to assess the impact of canine angulation on the severity of the resorption since this parameter was not evaluated in all selected studies. Early extraction of the primary canines is suggested in order to assist with the eruption of the palatally displaced permanent canines [36].

The diagnostic performance in CBCT regarding the severity of RR might depend on the different parameter settings of the machines used [30]. The variance regarding the incidence of RR found in the present study may be partially explained by the different CBCT systems used in the eligible studies. In vitro research proved that root resorption scores between different CBCT systems may differ to a statistically significant degree [35,37]. Different voxel sizes may affect the detectability of initial or slight RR [30]. A recent systematic review concluded that CBCT images with a voxel size of 0.20 mm might be unable to identify RR of a small magnitude and that studies with a voxel size ≤0.20 mm report significantly greater RR compared to studies with greater voxel sizes. As a result, smaller voxel sizes might be preferable to accurately diagnose RR but the benefit/risk ratio should be always considered prior to increasing the radiation dose [38]. Moreover, artifacts in CBCT images may affect the evaluation of resorption lacunae and should be avoided where possible [35]. Considering the diagnostic capability offered by CBCT, especially in cases of impacted teeth, maxillary hypoplasia, and orthognathic surgery, Portelli et al. compared standard- and low-dose CBCT protocols for orthodontic diagnosis and concluded that low-dose settings should be preferred in orthodontic practice, as they provide a significantly lower radiation dose to the patients ensuring good image quality [39].

The strengths of the present systematic review include a methodology following clear-cut guidelines. The search strategies were meticulously applied, covering electronic and written literature. Their character was comprehensive, including every available study in English. Additionally, an effort was made to minimize methodological bias. Screening, verification of eligibility, abstraction of information, assessment of the risk of bias, and quality of evidence were all performed in duplicate and any disagreement was resolved by discussion or consultation until a final consensus was achieved.

Nevertheless, a potential source of bias in the present review could be the inclusion of articles written only in English. There are also some limitations in this study, arising mainly from the nature and the characteristics of the data retrieved during the review process. All the included studies were non-randomized trials. Additionally, the limitations of this research, as well as most meta-analyses, relate to difficulties in the sample selection of eligible clinical studies, which render the comparison between results difficult. These articles display a degree of methodological heterogeneity related to the allocation of the participants among the treatment arms, a fact that may have a significant influence on treatment effects. Differences in the sample regarding the genetic background and gender selection may explain the excess variance between the studies. Therefore, further CBCT studies with more homogeneous groups that should additionally consider the risk factors related to maxillary canine impaction should be conducted.

## 5. Conclusions

Patients with impacted canines present a high frequency of lateral incisor root resorption, frequently to a mild degree.

The lateral incisors in the apical and middle thirds of the root are affected more frequently.

The high variance regarding the prevalence of lateral incisor root resorption among the selected studies stresses the need for further research.

The limitations of this systematic review relate to difficulties in the sample selection of eligible clinical studies.

Additional CBCT studies with more homogeneous groups that should additionally consider the risk factors related to maxillary canine impaction should be conducted.

## Figures and Tables

**Table 1:** The demographics of participants in the included studies [26,27,28,29,30,31,32].

Study	Ali et al., 2021	Dogramaci et al., 2015	Hadler-Olsen et al., 2015	Lai et al., 2012	Liu et al., 2008	Oberoi & Knueppel, 2012	Rafflenbeul et al., 2019
CBCT acquisitions	CBCT: CS 9300 3D (Carestream Dental LLC., Atlanta, GA, USA), FOV: 8 × 8, 80 kVp, 10 mA, and 20 s.	CBCT: Accuitomo 80 (Morita, Osaka, Japan), 70–90 kV, 3.0–4.0 mA, FOV: 40 × 40 mm or 60 × 60 mm, 17.5 s	CBCT: SCANORA 3D (Soredex, Charlotte, NC, USA), FOV: 6 × 6 cm and 7.5 × 10 cm, 85 kV, 45 mAs,	CBCT: Accuitomo 3D (Morita, Osaka, Japan), FOV: 4 × 4, 6 × 6, 8 × 8 cm, Voxels: 0.08 mm, 80 kV, 5.0 mA	CBCT: QR-DVT 9000 (NewTom, Verona, Italy)	CBCT: Mercury (Hitachi, Tokyo, Japan), 120 kVp, 15 mA, FOV: 12 inches	CBCT: NewTomTM VGi unit (QR s.r.l., Verona, Italy), FOV 8 8, 100 kV, VOXEL SIZE 150 μm 3.6–5.4 s
Total N	41	85	37	113	175	29	60
Ethnicity	Multicultural	Multicultural	Caucasian	Caucasian	Multicultural	Multicultural	Caucasian
Males	9	25	15	39	55	7	26
Females	32	60	22	74	120	22	34
Age mean	20.8	18.1	11.9	19.35	16.9	16.6	12.2
Age SD	11.1	10.3	9.26	13.65	6.9	9.26	1.9
Total impacted	56	110	46	134	210	42	83
Bilateral	30	50	24	42	70	26	46
Unilateral	26	60	22	92	140	16	37
Total males impacted	12	25			43		
Bilateral males	3	8			24		
Unilateral males	6	17			67		
Unilateral males Left		6					
Unilateral males Right		11					
Total females impacted	44	60			143		
Bilateral females	12	17			46		
Unilateral females	20	43			97		
Unilateral females-left		17					
Unilateral females-right		26					
Root resorption (%)	41	10.09	60.87	35.82	75	40.48	55.7
Mild	85.71	50	67.86	39.58	46.67	64.71	81.8
Moderate	9.52	20	14.29	12.5	31.43	23.53	15.9
Severe	4.76	30	17.86	47.92	21.9	11.76	2.3
Cervical		8	4	2			2
Middle		30	31	22			22
Apical		66	42	24			17

CBCT: Cone-beam computed tomography.

**Table 2:** Risk of bias in individual studies with ROBINS-I assessment tool [26,27,28,29,30,31,32].

Study	Bias Due to Confounding	Bias in Selection of Participants into the Study	Bias in Classification of Interventions	Bias Due to Deviations from Intended Intervention	Bias Due to Missing Data	Bias in Measurement of Outcomes	Bias in Selection of the Reported Results	Overall
Ali et al., 2021	Moderate	Low	Moderate	Low	Low	Moderate	Low	Moderate
Dogramaci et al., 2015	Moderate	Low	Moderate	Low	Low	Moderate	Low	Moderate
Hadler-Olsen et al., 2015	Moderate	Low	Moderate	Low	Low	Moderate	Low	Moderate
Lai et al., 2012	Moderate	Low	Moderate	Low	Low	Moderate	Low	Moderate
Liu et al., 2008	Moderate	Low	Moderate	Low	Low	Moderate	Low	Moderate
Oberoi & Knueppel, 2012	Moderate	Low	Moderate	Low	Low	Moderate	Low	Moderate
Rafflenbrul et al., 2019	Moderate	Low	Moderate	Low	Low	Moderate	Low	Moderate

## Data Availability

The dataset used or analyzed during the proposed systematic review will be available from the corresponding author on reasonable request.

## References

[1] Al-Zoubi H., Alharbi A.A., Ferguson D.J., Zafar M.S. (2017). Frequency of impacted teeth and categorization of impacted canines: A retrospective radiographic study using orthopantomzgrams. Eur. J. Dent..

[2] Aydin U., Yilmaz H.H., Yildirim D. (2004). Incidence of canine impaction and transmigration in a patient population. DentoMaxilloFacial Radiol..

[3] Celikoglu M., Kamak H., Oktay H. (2010). Investigation of transmigrated and impacted maxillary and mandibular canine teeth in an orthodontic patient population. J. Oral Maxillofac. Surg..

[4] Ericson S., Kurol J. (1986). Radiographic assessment of canine eruption in children with clinical signs of eruption disturbances. Eur. J. Orthod..

[5] Ericson S., Kurol J. (1987). Incisor resorption caused by maxillary cuspids. A radiographic study. Angle Orthod..

[6] Rózsa N., Fábián G., Szádeczky B., Kaán M., Gábris K., Tarján I. (2003). Prevalence of impacted permanent upper canine and its treatment in 11-18-year-old orthodontic patients. Fogorv. Sz..

[7] Kumar S., Mehrotra P., Bhagchandani J., Singh A., Garg A., Kumar S., Sharma A., Yadav H. (2015). Localization of impacted canines. J. Clin. Diagn. Res..

[8] Grover P.S., Lorton L. (1985). The incidence of unerupted permanent teeth and related clinical cases. Oral Surg. Oral Med. Oral Pathol..

[9] Shah R.M., Boyd M.A., Vakil T.F. (1978). Studies of permanent tooth anomalies in 7886 Canadian individuals. II: Congenitally missing, supernumerary and peg teeth. Dent. J..

[10] Bishara S.E. (1992). Impacted maxillary canines: A review. Am. J. Orthod. Dentofacial. Orthop..

[11] Ristaniemi J., Rajala W., Karjalainen T., Melaluoto E., Iivari J., Pesonen P., Lähdesmäki R. (2022). Eruption pattern of the maxillary canines: Features of natural eruption seen in PTG at the late mixed stage-Part I. Eur. Arch. Paediatr. Dent..

[12] Mohammed A.K., Sravani G., Vallappareddy D., Rao A.R., Qureshi A., Prasad A.N. (2020). Localization of Impacted Canines—A Comparative Study of Computed Tomography and Orthopantomography. J. Med. Life..

[13] Peck S., Peck L., Kataja M. (1994). The palatally displaced canine as a dental anomaly of genetic origin. Angle Orthod..

[14] Ericson S., Kurol P.J. (2000). Resorption of incisors after ectopic eruption of maxillary canines: A CT study. Angle Orthod..

[15] Alqerban A., Jacobs R., Lambrechts P., Loozen G., Willems G. (2009). Root resorption of the maxillary lateral incisor caused by impacted canine: A literature review. Clin. Oral Investig..

[16] Ericson S., Kurol J. (1988). Resorption of maxillary lateral incisors caused by ectopic eruption of the canines. A clinical and radiographic analysis of predisposing factors. Am. J. Orthod. Dentofac. Orthop..

[17] Ericson S., Bjerklin K., Falahat B. (2002). Does the canine dental follicle cause resorption of permanent incisor roots? A computed tomographic study of erupting maxillary canines. Angle Orthod..

[18] Guarnieri R., Cavallini C., Vernucci R., Vichi M., Leonardi R., Barbato E. (2016). Impacted maxillary canines and root resorption of adjacent teeth: A retrospective observational study. Med. Oral Patol. Oral.

[19] Westphalen V.P., Gomes de Moraes I., Westphalen F.H., Martins W.D., Souza P.H. (2004). Conventional and digital radiographic methods in detection of simulated external root resorptions: A comparative study. Dentomaxillofac. Radiol..

[20] Tsolakis A.I., Kalavritinos M., Bitsanis E., Sanoudos M., Benetou V., Alexiou K., Tsiklakis K. (2018). Reliability of different radiographic methods for the localization of displaced maxillary canines. Am. J. Orthod. Dentofacial. Orthop..

[21] Liberati A., Altman D.G., Tetzlaff J., Mulrow C., Gøtzsche P., Ioannidis, Clarke M., Devereaux P.J., Kleijnen J., Moher D. (2009). The PRISMA statement for reporting systematic reviews and meta- analyses of studies that evaluate healthcare interventions: Explanation and elaboration. BMJ.

[22] Sterne J.A., Hernán M.A., Reeves B.C., Savović J., Berkman N.D., Viswanathan M., Henry D., Altman D.G., Ansari M.T., Boutron I. (2016). ROBINS-I: A tool for assessing risk of bias in a non-randomised studies of interventions. BMJ.

[23] DerSimonian R., Laird N. (1986). Meta-analysis in clinical trials. Control Clin. Trials.

[24] Nyaga V.N., Arbyn M., Aerts M. (2014). Metaprop: A Stata command to perform meta-analysis of binomial data. Arch. Public Health.

[25] Higgins J.P., Thompson S.G., Deeks J.J., Altman D.G. (2003). Measuring inconsistency in meta-analyses. BMJ.

[26] Ali I.H., Al-Turaihi B.A., Mohammed L.K., Alam M.K. (2021). Root Resorption of Teeth Adjacent to Untreated Impacted Maxillary Canines: A CBCT Study. Biomed. Res. Int..

[27] Rafflenbeul F., Gros C.I., Lefebvre F., Bahi-Gross S., Maizeray R., Bolender Y. (2019). Prevalence and risk factors of root resorption of adjacent teeth in maxillary canine impaction, among untreated children and adolescents. Eur. J. Orthod..

[28] Doğramacı E.J., Sherriff M., Rossi-Fedele G., McDonald F. (2015). Location and severity of root resorption related to impacted maxillary canines: A cone beam computed tomography (CBCT) evaluation. Aust. Orthod. J..

[29] Hadler-Olsen S., Pirttiniemi P., Kerosuo H., Limchaichana N.B., Pesonen P., Kallio-Pulkkinen S., Lähdesmäki R. (2015). Root resorptions related to ectopic and normal eruption of maxillary canine teeth—A 3D study. Acta Odontol. Scand..

[30] Lai C.S., Bornstein M.M., Mock L., Heuberger B.M., Dietrich T., Katsaros C. (2013). Impacted maxillary canines and root resorptions of neighbouring teeth: A radiographic analysis using cone-beam computed tomography. Eur. J. Orthod..

[31] Liu D.G., Zhang W.L., Zhang Z.Y., Wu Y.T., Ma X.C. (2008). Localization of impacted maxillary canines and observation of adjacent incisor resorption with cone-beam computed tomography. Oral Surg. Oral Med. Oral Pathol. Oral Radiol. Endod..

[32] Oberoi S., Knueppel S. (2012). Three-dimensional assessment of impacted canines and root resorption using cone beam computed tomography. Oral Surg. Oral Med. Oral Pathol. Oral Radiol..

[33] Schroder A.G.D., Guariza-Filho O., de Araujo C.M., Ruellas A.C., Tanaka O.M., Porporatti A.L. (2018). To what extent are impacted canines associated with root resorption of the adjacent tooth?: A systematic review with meta-analysis. J. Am. Dent. Assoc..

[34] Kiljunen T., Kaasalainen T., Suomalainen A., Kortesniemi M. (2015). Dental cone beam CT: A review. Phys. Med..

[35] Alqerban A., Jacobs R., Fieuws S., Nackaerts O., Willems G., The SEDENTEXCT Project Consortium (2011). Comparison of 6 cone-beam computed tomography systems for image quality and detection of simulated canine impaction-induced external root resorption in maxillary lateral incisors. Am. J. Orthod. Dentofacial Orthop..

[36] Almasoud N.N. (2017). Extraction of primary canines for interceptive orthodontic treatment of palatally displaced permanent canines: A systematic review. Angle Orthod..

[37] Alqerban A., Jacobs R., Souza P.C., Willems G. (2009). In-vitro comparison of 2 cone-beam computed tomography systems and panoramic imaging for detecting simulated canine impaction-induced external root resorption in maxillary lateral incisors. Am. J. Orthod. Dentofac. Orthop..

[38] Samandara A., Papageorgiou S.N., Ioannidou-Marathiotou I., Kavvadia-Tsatala S., Papadopoulos M.A. (2018). Evaluation of orthodontically induced external root resorption following orthodontic treatment using cone beam computed tomography (CBCT): A systematic review and meta-analysis. Eur. J. Orthod..

[39] Portelli M., Militi A., Lo Giudice A., Lo Giudice R., Fastuca R., Ielo I., Mongelli V., Giudice G.L., Martintoni A., Manuelli M. (2018). Standard and low-dose cone beam computer tomography protocol for orthognatodontic diagnosis: A comparative evaluation. J. Biol. Regul. Homeostatic. Agents..

